# Design Principles of a Genetic Alarm Clock

**DOI:** 10.1371/journal.pone.0047256

**Published:** 2012-11-07

**Authors:** Jaroslav Albert, Marianne Rooman

**Affiliations:** BioModeling, BioInformatics and BioProcesses Department, Université Libre de Bruxelles, Bruxelles, Belgium; Baylor College of Medicine, United States of America

## Abstract

Turning genes on and off is a mechanism by which cells and tissues make phenotypic decisions. Gene network motifs capable of supporting two or more steady states and thereby providing cells with a plurality of possible phenotypes are referred to as genetic switches. Modeled on the bases of naturally occurring genetic networks, synthetic biologists have successfully constructed artificial switches, thus opening a door to new possibilities for improvement of the known, but also the design of new synthetic genetic circuits. One of many obstacles to overcome in such efforts is to understand and hence control intrinsic noise which is inherent in all biological systems. For some motifs the noise is negligible; for others, fluctuations in the particle number can be comparable to its average. Due to their slowed dynamics, motifs with positive autoregulation tend to be highly sensitive to fluctuations of their chemical environment and are in general very noisy, especially during transition (switching). In this article we use stochastic simulations (Gillespie algorithm) to model such a system, in particular a simple bistable motif consisting of a single gene with positive autoregulation. Due to cooperativety, the dynamical behavior of this kind of motif is reminiscent of an alarm clock – the gene is (nearly) silent for some time after it is turned on and becomes active very suddenly. We investigate how these sudden transitions are affected by noise and show that under certain conditions accurate timing can be achieved. We also examine how promoter complexity influences the accuracy of this timing mechanism.

## Introduction

Genetic circuits bear resemblance to human-made (e. g. electrical) circuits, in that both types perform a specific function or functions and are optimized to be robust against stochastic fluctuations and, in the former case, genetic mutations. However, the natural optimization of the genetic circuits seems yet incomplete and in constant flux. Using such naturally occurring circuits, synthetic biologists make improvements where nature fell short as well as devise new and novel motifs previously unseen.

Designing genetic circuits has been a major preoccupation by researchers working in the field of synthetic biology. Thus far, the record of successfully designed and implemented biological systems is noteworthy and still growing. Examples of synthetically constructed systems include: the toggle switch [1], positive autoregulation motifs [2], gene networks for tuning protein degradation [3], complex promoters [4] and many others (see [5], [6] and references therein). In order for this trend of success to continue, it is imperative that both, theoretical modeling and experimentation, continue to refine existing designs as well as invent and test new ones.

Network motifs with positive autoregulation have been studied extensively [7]–[10] and their functions are well-known: (i) they slow the response time to stimuli, (ii) they increase the intrinsic noise and hence variability among a cell population, and (iii) those capable of supporting more than one steady state can function as bistable switches. In some cases these functions work together as, for example, during an epigenic differentiation where the intrinsic noise can trigger a random transition from low to high protein concentration, hence giving rise to two different populations of cells [11]–[13]. In other cases the delayed response serves the purpose of filtering short noisy bursts.

Longer delays – several hundred minutes or more – have been observed in real biological systems as in, for example, certain genetic circuits that control cell death [14]. Such delay-generating circuits usually involve motifs containing several genes, which makes them less ideal as systems to emulate by synthetic biology. On the other hand, due to greater degree of freedom and parameter space, circuits comprised of several genes tend to be more robust against external fluctuations and genetic mutations. Somewhere between this practical drawback and functional advantage lies an optimal design for generating controlled delayed responses.

Our aim here is to model, using stochastic simulation, a bistable gene switch capable of behaving like an alarm clock and discover general design principles that would facilitate its construction. More specifically, we want to know what makes the time of switching predictable to a high degree of accuracy. Nature gives us examples of breathtaking accuracy, e. g. in multicellular organisms which, during gestation, follow a temporal and spacial pattern so predictable “it could be used to set a watch” [15]. This observation inspired us to hope that accuracy in the system at hand was not asking too much.

To narrow the focus of our study, we set out to answer these three specific questions: (i) Is accurate switching at all possible in this type of system? (ii) What effects, if any, does the length of the delay have on this accuracy? (iii) What are the conditions under which this accuracy is possible?

## Results

### 0.1 Intrinsic properties of positive feedback

#### 0.1.1 Delayed response to an external input

**Figure 1:**
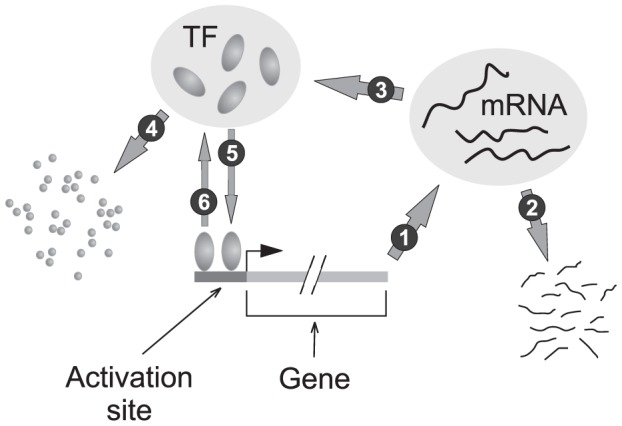
Schematic diagram of the gene network. The evolution of mRNA and protein numbers are governed by the six processes shown: 1: Transcription; 2: mRNA degradation; 3: Translation; 4: Protein degradation; 5: TF-promoter associations; and 6: TF-promoter disassociation. Binding of 2 TFs to the promoter (activation site) enhances the transcription which in turn increases the rate of TF-promoter association.

**Figure 2:**
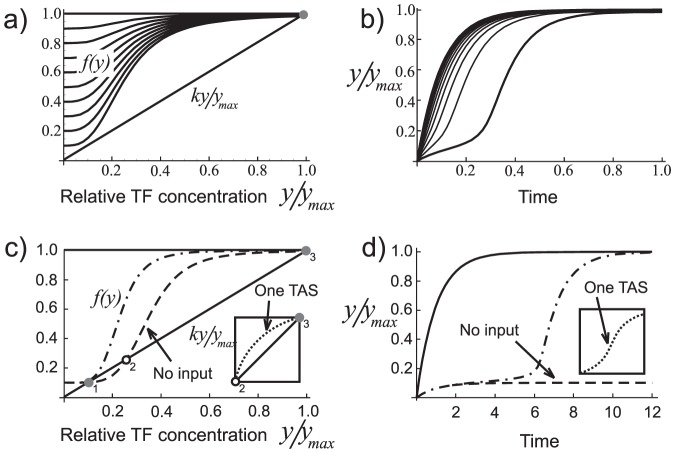
Dynamical properties a system with positive feedback. a) Plot of TF degradation rate, 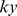 (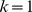) and TF production rate 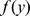 as a function of 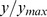 – the fraction of TF concentration relative to its final steady state value. The difference 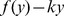 is the net production. b) Plot of TF concentration as a function of time. c) A gene switch in an on (off) postion when (no) input is present: dot-dashed line (dashed line). d) Evolution of TF concentration for 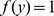 (solid line), with no input (dashed line), and with input (dot-dashed line). The small frame in (c) and (d) shows the situation for a single TAS.

In systems with autoregulation, the proteins encoded by a gene themselves regulate their production rate; they are referred to as transcription factors (TF). The dynamical behavior of TF concentration, from now on denoted as 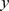, is shaped by two forces: production and degradation. In most cases, the degradation term is linearly dependent on 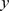, whereas the rate of production is generally a more complicated function of 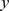, *e. g.* a Hill function, 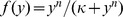, where 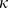 is called the Hill coefficient and is related to 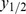, the TF concentration at which 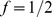; 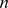 is the Hill exponent, an integer determined by the promoter complexity. Figure (1) is an illustration of a promoter with two transcription activation sites (TAS) to which only these specific TFs can bind; this gives rise to 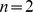. Figure (2a) shows graphically the dependence of degradation on the TFs, 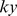, and several curves representing different production rates, 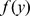. The important feature to notice is the reduced difference between 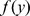 and 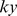 as compared to the case of constant production rate, 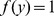. This means that systems with positive feedback will always take longer to reach their steady state than those without, provided their steady states are the same. Figure (2b) shows the TF concentration dynamics for each case of 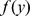 in (2a) governed by the equation 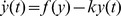.

#### 0.1.2 Bistability and long delays

When more than one TF is required for transcription initiation, the system may have more than one stable steady state and can be induced to evolve from one to another by changing one or more of its parameters (reaction rates). Among these, the one that most commonly occurs in nature is the bistable system [16]. In figure (1d), the curve on the right represents a bistable configuration: the system rests indefinitely in either of the two stable steady states, 1 or 3 (point 2 is unstable), corresponding to 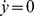. If the system starts out at point 1, it will remain there until an external input, *e. g.* external chemical, change in environmental conditions (temperature, pH, light) or a TF from a different gene, modifies one or more of the system parameters and hence the curve in such a way that point 3 becomes the only available steady state. Figure (2d) illustrates the dynamics of this arrangement (the point-dashed curve). The initial rate of net TF production depends on the difference along the vertical between the curve 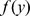 and the degradation line, 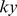. In principle, this difference can be arbitrarily small, making the system linger near point 1 for an arbitrarily long time.

We should point out the fact that multi-stability can only be achieved for 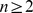. This can be seen in the rectangle within the graph of Fig. (2c), showing 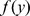, for which 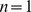, and 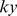. Notice that only points 2 and 3 are present, 3 being the only stable one. Dynamics of 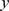 for this system are shown in the rectangle within Fig. (2d).

#### 0.1.3 Switching in the presence of noise

When noise is taken into account, the situation becomes more complicated. If we define the delay as the time when the TFs reach one third of their final steady state concentration (this definition is arbitrary), we should expect to find a distribution of delays centered near the value predicted by deterministic models.

**Figure 3:**
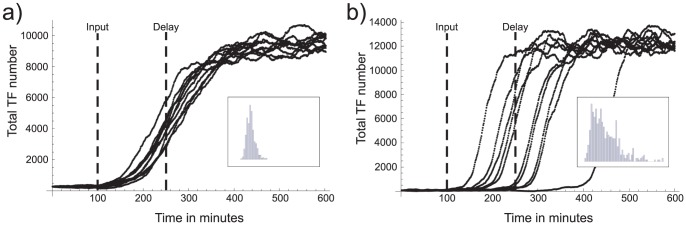
Simulation of TF evolution. A unique input was introduced at 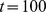 min for each case so as to generate a delay of 250 min. While for clarity only 10 runs are shown, the delay distribution (shown in the boxes) was computed from 100 runs. A clear difference in delay distribution can be seen between (a) and (b). The parameter values in inverse minutes are: in a) transcription rate 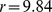, translation rate 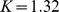, TF association rate 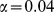, TF dissociation rate, 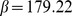 mRNA degradation rate 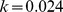, and protein degradation rate 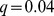; and in b) 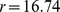, 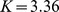, 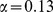, 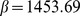, 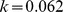, and 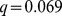.

Though noise will always be present, there can be significant differences in delay uncertainty between systems with similar averaged dynamics but different parameter values. Figure (3a–b) shows a simulation of two such systems, using the Gillespie algorithm (see section “Methods”). At time t = 0 the promoter binding rate of each system was increased by such amount that the averaged delay (as defined above) was very similar 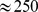 min. One can see that while their average profiles are similar, apart from their final steady state values, their delay distribution is quite different. In the following sections we investigate the source of this difference.

### 0.2 Taming the noise: how to construct a switch with predictable delays

#### 0.2.1 Deterministic Model

To go beyond the qualitative description of the system at hand and understand its dynamical behavior in terms of chemical reactions, we must include in our model the interactions of the TFs with their TAS (transcription activation sites). The rate equations then read:
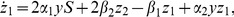
(1)

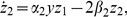
(2)

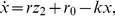
(3)

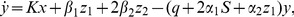
(4)

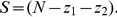
(5)The variables 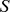, 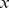 and 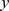 represent the concentrations of the free TAS, mRNA and TF respectively; the other variables, 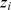, signify the concentrations of complexes made up of 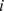 TFs and 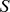. The quantity 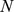 stands for total DNA copy number; here, it is set to 1. The parameters 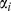 and 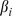 denote the rate of association and dissociation between TF and the activation site respectively. One can write 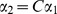 where 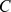 is the cooperativity factor. When no cooperativity is present, 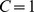, we have 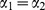 and 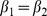; in all that follows (unless noted otherwise), we will consider only this case, setting 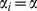 and 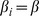. The other parameters, 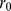, 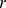, 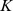, 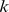, 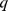 denote, in the respective order, the rates for: basal transcription (when 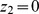), maximal transcription (when 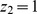), translation, mRNA degradation, and TF degradation. The factor of 2 appearing in Eqs. (1)–(4) comes from the fact that formation of 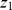 and dissociation of 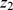 can happen in two distinctive ways: TF can bind to one or the other TAS; and, similarly, when two TFs are bound to 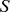, each has an opportunity to escape. In writing Eq. (3) we assumed that only by forming complex 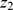 does the transcription rate increase from its basal value 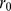 to 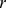.

For the purpose of unifying our graphical representations of Fig. (2a–d) and the model we have just defined, one may decouple Eqs. (1) and (2) from the others by setting Eqs. (2) and (3) to zero and solving for 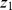 and 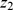 in terms of 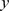:
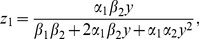
(6)

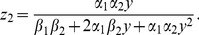
(7)What allows this approximation is the (experimental) observation that mRNA and TFs take several orders of magnitude longer to reach equilibrium than 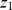 and 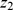. Further steps can be taken by expressing 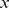 and 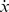 in terms of 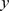 and 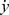, differentiating Eq. (4), and inserting to it the newly expressed 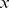 and 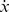. This leads to:
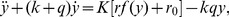
(8)where
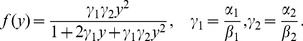
(9)With the second derivative in Eq. (8) it is now easy to interpret the right hand side as a force. For the case 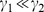, the function 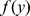 acquires its Hill form discussed earlier with 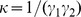.

Note that while the profile of 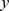 as a solution of Eq. (8) is only approximately equal to that which is a solution of the full system, (1–4)), their fixed points 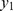, 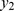 and 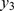, and 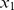, 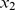 and 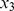 are identical.

#### 0.2.2 Exploring the parameter space

Once a deterministic model is defined, its dynamical properties can be explored as a function of its parameters. Here we are interested in a specific dynamical behavior, namely, delayed response to an external input. We imagine that the switch is in an off position, *i. e.* the lowest fixed point, when the input is introduced. The input may be a signaling molecule which can, in principle, depending on its type, change the value of any system parameter, or even several of them. For our study we chose 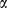 to be the control parameter. This is a reasonable choice as in the real systems, *e. g. E. coli*, many types of signaling molecules can change the affinity between TFs and their TAS. Thus, when the input is zero, the affinity of transcription factor 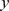 to bind its activation region is such that the system has three fixed points (see Figure (2c)). Once the input is introduced the TFs undergo a conformational change that allows them to bind more strongly to their activation sites. This shifts the curve in Fig. (2c) to the left, leaving only the third fixed point available.

In order to understand how the delay uncertainty depends on the system parameters, we first selected 275 different parameter sets, each satisfying the following constraints: 1) the parameter values were restricted to a realistic range (see the “Methods section”); 2) only those parameter sets for which 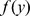 had three positive roots (fixed points) were considered; 3) a lower bound was placed on the possible values of the first fixed point 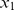 (no such bound was imposed on 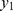), and was increased incrementally after every parameter set selection; this ensured that large values of 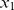 were also selected; in the present case the range we chose 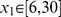 (the reason for this choice is explained later in the section) 4) the distance between points 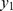 and 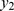 was kept smaller than that between 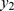 and 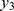; without this constraint the dynamical behavior would not resemble the switch-like profile of Fig. (2d) (dot-dashed line); and, lastly, 5) only those parameter sets for which the distance between 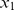 and 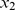 was larger than 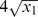 were kept; this constraint served as insurance that the tail of the probability distribution of mRNA falls off to zero before it reaches the second fixed point, *i. e.* its variance is four times smaller than the distance between 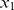 and 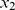, where the variance is taken as 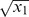, the true value for constant transcription rate [17]; this, of course, is only an approximation, as the dependence of 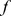 on 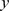 is negligible only up to certain values of 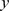.

Next, in each set we increased 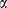 by such a factor that the numerical solution of Eqs. (1–5) yielded a delayed response of 300 (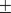
5) minutes.

Once the parameter sets were selected in this manner, we performed a stochastic simulation, using the Gillespie algorithm [18], 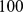 times for each set. In each run, we started with 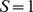, 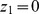, 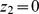, 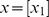 and 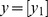 at time 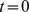, where 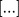 means rounded to the nearest integer, and let the system evolve to its equilibrium. When the input was introduced, we set the time to zero again. Each run was terminated when the protein number exceeded one half of its value at the final steady state; the time of termination – the delay – was recorded.

We repeated the above procedure with parameter sets in which all but the value of 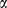
s were the same. The new 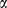
s were chosen so as to obtain delays of 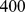 minutes. In order to determine what effect, if any, does promoter complexity have on the accuracy of delays, we derived equations similar to Eqs. (1)–(5) for three TAS, instead of two, and repeated the procedure described above.

**Figure 4:**
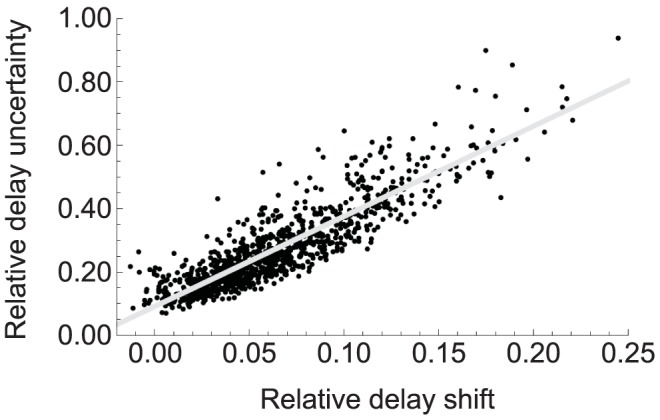
Relative delay shift vs. relative delay uncertainty. This scatter plot indicates a linear relationship between the relative delay uncertainty 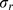 and the relative difference of the observed average and deterministic delay 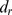. From an engineering point of view, this tells us that if one manages to reduce 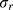, 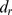 is likely to be reduced as well.

**Figure 5:**
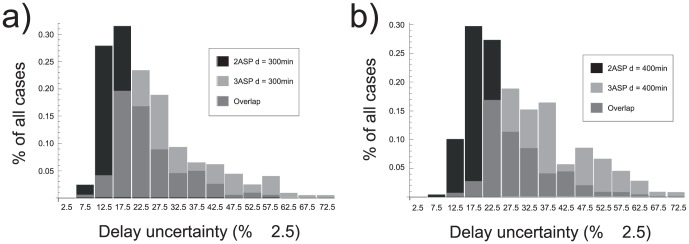
Uncertainty distributions. Distribution of relative delay uncertainty 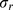 for 2 and 3 TAS for a delay of 300 min (a) and 400 min (b). Each bar gives the fraction of all cases with 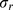 between the values indicated on the x-axis 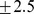 in %.

Based on our results we observed several trends. First, the delays predicted by Eqs. (1)–(5) are almost always less than the averaged delays obtained from the stochastic simulations. Second, the relative delay shifts and delay uncertainties, defined by
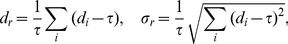
(10)where 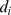 is the delay of the 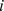
th run and 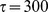 or 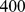 minutes, approximately follow a linear trend as shown in Fig. (4); this implies that the more the averaged delay differs from the deterministic delay, the greater the delay uncertainty. Another trend can be seen in Fig. (5a–b) where we plotted the fraction of those cases whose 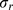 falls between the range of values indicated on the x-axis for both, the two and three TAS. From these, one can draw two conclusions: transcription initiation requiring high number of activator sites tends to lead to less accurate delays; and, the longer the delay is the less accurate it is.

### 0.3 Correlation between the delay uncertainties and system parameters

**Figure 6:**
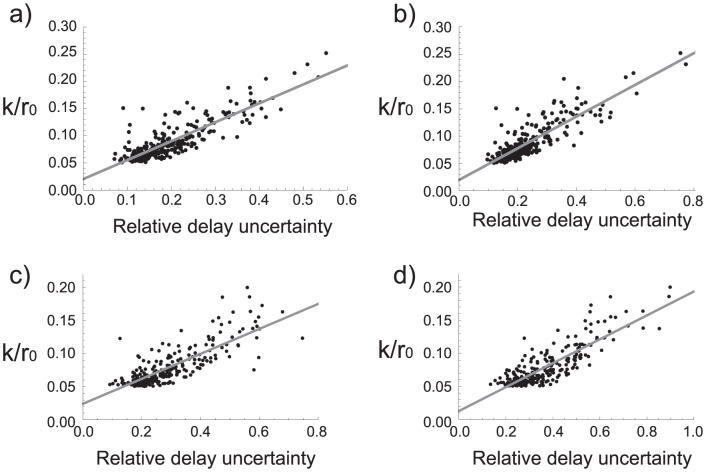
Relative delay uncertainty vs. G. Scatter plot of the relative delay uncertainty 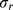 and the function of 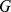 (Eq. (13)) for a) 2 TAS, 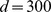 min; b) 2 TAS, 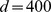 min; c) 3 TAS, 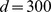 min; and d) 3 TAS, 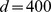 min.

Since the system parameters completely define a model, any stochastic quantity can, in principle, be calculated in terms of them, whether one uses a stochastic algorithm or the master's equation. This is also true for 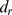 and 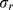. However, obtaining an analytical expression for, say, 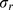 as a function of the system parameters is not feasible. The most one can do is find a trend between some function of the parameters and 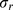. Earlier we hypothesized that the initial mRNA number, which is approximately given by 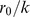, is a factor in determining 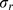. Figure (6a–d) shows that this trend indeed exists; however, the fact that many points fall far off the fitting line implies that the story is more complicated and requires cooperation of the other parameters.

**Figure 7:**
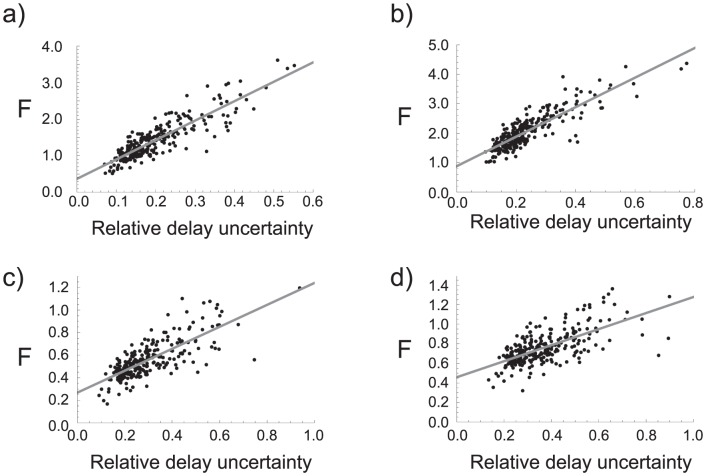
Relative delay uncertainty vs. F. Scatter plot of the relative delay uncertainty 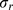 and the function of 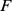 (Eq. (12)) for a) 2 TAS, 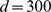 min; b) 2 TAS, 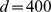 min; c) 3 TAS, 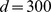 min; and d) 3 TAS, 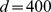 min.

**Figure 8:**
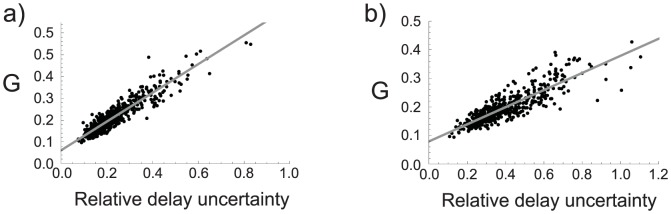
Relative delay uncertainty vs. G. Scatter plot of the relative delay uncertainty 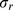 and the function of 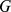 (Eq. (13)) for a) 2 TAS, 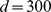 min and 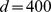 min; b) 3TAS, 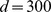 min and 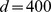 min.

To obtain a more accurate relation between 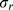 and the system parameters we took an ansatz
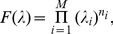
(11)with 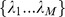 being the set of system parameters and 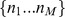 a set of integers, such that 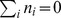 (since zero is the dimension of 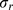). The best fit is given by
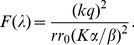
(12)
Figure (7a–d) shows the plot of the correlation between 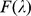 and 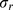. As a final step, we searched for an optimal linear combination of 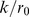 and 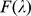 and found that
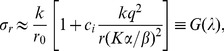
(13)with 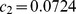 for two TAS and 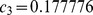 for three TAS, gives the best fit, which can be seen in Fig. (8a–b).

While Eq. (13) does not provide an exact relation, it does shed light on the condition that needs to be satisfied in order to construct a switch with accurate delays, namely that 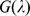 must be small. A more general approach, one that does not depend on the coefficient 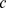, would be to require that 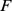 be as small as possible while keeping 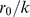 large. To put this into a test, we generated one hundred parameter sets for two TAS and kept the first five for which 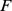 was the smallest; the same was done on the opposite end, corresponding to the largest 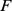. For all ten sets, 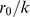 was kept above 20. Performing the same stochastic simulations as before showed that the first group, with small 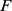
s, all had 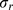 below 0.1 (10%), while the latter averaged at 18%. We repeated this procedure, again for two TAS with cooperativity, 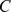,equal to 2,3,4 and 5 (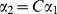). Table 1 presents the average scores and deviations for all ten cases. Regardless of the cooperativity, all five cases show significant discrepancy in 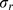 between low and high values of 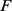.

**Table 1:** Delay uncertainty predictions.

C	Low F: 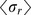	Low F: varience	High F: 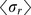	High F: Varience
1	8.01	2.39	18.50	3.51
2	11.30	0.59	20.42	2.21
3	10.04	0.80	20.73	2.41
4	11.14	0.71	18.98	2.70
5	12.49	1.88	23.49	2.50

Results of predicted relative delay uncertainties 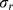 and their variances for different coopereativities 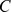.

## Discussion

In the introduction we posed three specific questions: (i) Is accuracy in delayed gene switching achievable in the system at hand? (ii) What effect, if any, does the length of the delay have on the delay's accuracy? and (iii) What are the conditions that allow this type of switch to generate predictable delays? Our study shows that it is indeed possible to have accurate delays under certain conditions. With regards to the second question, we found that the relative variance of the delays is sensitive to the delay's length: the longer the delay, the greater the variance tends to be. And finally, in order to answer the third question, we have derived approximate phenomenological relations between the system parameters and the relative delay uncertainties for two and three TAS and demonstrated that, although these relations are not exact, they can be reliable in distinguishing systems which support accurate delays from those that do not.

The reader should keep in mind that all our results were based on a simple stochastic model that ignored all other sources of noise, e. g. basal TF complex formation, mRNA splicing, etc. However, simple models have proven to be useful in the past and can provide insight into general properties of real systems. By virtue of its simplicity, our model system can be constructed in simple organisms such as *E. coli* [19] and the aforementioned results can be verified.

## Materials and Methods

Each parameter was assigned a random value restricted to a range based on several experimental sources [20]–[22] (and references therein).
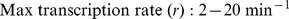


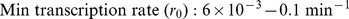


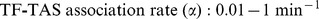


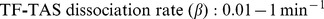


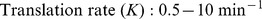


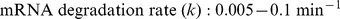


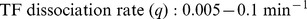
All simulations were done using the Gillespie algorithm [18] in Mathematica. The chemical reactions for the two TAS are:
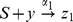


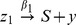


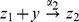


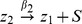


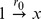


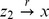


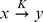


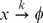


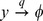
where 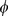 stands for degradation.

Optimization of the parameters 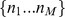, Eq. (11) (there were constrained to be integers) was done using the default global minimization algorithm in Mathematica. The fitting parameters, 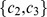, Eq. (13), were calculated analytically by minimizing the distance function
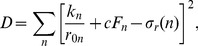
(14)for the case of two and three TAS, the result of which is
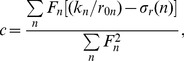
(15)where 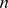 labels a particular parameter set.
